# Faces in Event Streams (FES): An Annotated Face Dataset for Event Cameras

**DOI:** 10.3390/s24051409

**Published:** 2024-02-22

**Authors:** Ulzhan Bissarinova, Tomiris Rakhimzhanova, Daulet Kenzhebalin, Huseyin Atakan Varol

**Affiliations:** Institute of Smart Systems and Artificial Intelligence, Nazarbayev University, Astana 010000, Kazakhstan; ubissarinova@nu.edu.kz (U.B.); tomiris.khalimova@nu.edu.kz (T.R.); kenzhebalin.d@gmail.com (D.K.)

**Keywords:** computer vision, event camera, event stream, face detection, facial landmark detection

## Abstract

The use of event-based cameras in computer vision is a growing research direction. However, despite the existing research on face detection using the event camera, a substantial gap persists in the availability of a large dataset featuring annotations for faces and facial landmarks on event streams, thus hampering the development of applications in this direction. In this work, we address this issue by publishing the first large and varied dataset (Faces in Event Streams) with a duration of 689 min for face and facial landmark detection in direct event-based camera outputs. In addition, this article presents 12 models trained on our dataset to predict bounding box and facial landmark coordinates with an mAP_50_ score of more than 90%. We also performed a demonstration of real-time detection with an event-based camera using our models.

## 1. Introduction

Event-based imaging is considered a new paradigm in computer vision because of its distinct representation of visual data [1,2,3,4]. Event cameras use bioinspired vision sensors that record relative pixel-level intensity changes in a scene [5], as opposed to capturing frame-based intensity images. These illumination changes are referred to as events. An event is a four-dimensional tuple (x,y,p,t) that records a pixel-intensity change at the position (x,y) and the time *t*. The polarity p∈(0,1) is 0 for decreasing and 1 for increasing pixel-intensity changes. Event cameras do not rely on a global clock—that is, each pixel operates independently and is only recorded when a change in illuminance is detected. This allows for a very high temporal resolution of an event stream, which is usually measured in microseconds [1]. In general, event cameras offer significant advantages over conventional cameras, such as ultra-low latency, no-motion blur, low power consumption, and high dynamic range [4].

These properties make event cameras suitable for robotics and computer vision applications. Indeed, after the release of the first commercial event camera in 2008 [1], these systems found utilization in different settings. For example, an Internet of Things (IoT) crime detection application used an event camera to transmit data to a processing node only when an event was detected [6]. Reduced energy consumption and bandwidth thanks to the event camera made the IoT application more efficient, compared to traditional surveillance systems, which remain operational, regardless of whether activity is observed in the field of view. The absence of motion blur and the presence of high dynamic range enable event cameras to acquire reliable visual data during high-speed motion. In [7], these features of event cameras were integrated with frame-based imaging and depth images to address the challenges of low-light scenes and rapid motion aboard an autonomous quadrotor. In addition, in [8], event cameras were used in micro air vehicles to obtain optical flow information for navigation. Event cameras were also tested for daytime and nighttime traffic data collection, monitoring vehicle speeds in the range of 20 to 300 km/h, resulting in a near-zero mean error and a standard deviation of 2.3 km/h in speed estimation [9]. A recent work [10] has also shown that combined RGB and event based modalities achieve high accuracy in lip reading.

Despite numerous benefits and use cases, adoption of event cameras remains limited. This is likely due to the scarcity of datasets collected with event cameras and the deficiency of tools and algorithms for event-based processing, especially high-performance deep learning pipelines.

Facial images contain a wealth of features that are highly relevant to message transmission during intersocial communications [11]. Facial expressions convey information not only about a person’s emotional state, but also about cognitive human states, such as interest, boredom, confusion, and stress [12]. From the perspective of cognitive psychology, the face is a key distinctive feature in determining an individual’s identity. In this regard, the human cognitive system has evolved to extract structural codes from faces, thus acquiring the components of facial structures that enable us to distinguish between faces. In fact, there is a special region in the brain for processing visual stimuli on the face, the fusiform face area (FFA) [13].

As with the human cognitive system, faces play an important role in artificial intelligence applications. Face recognition systems are widely in service, primarily as a biometric tool for human identity validation [14], in access control systems to enable secure environments [15], and to determine the location of a missing person [16]. While face detection is used for video conferencing, crowd surveillance, intelligent human–computer interfaces, content-based image retrieval, and video coding [17], facial landmarks are used to create virtual avatars via head pose estimation [18] or even for diagnosing health disorders, such as fetal alcohol spectrum disorder [19].

**Figure 1:**
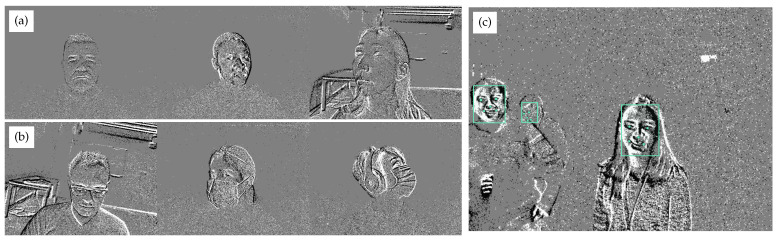
Snapshots from the dataset showing the diversity of subjects: (**a**) Participants of different ethnicities and both genders in the controlled environment, (**b**) participants wearing various accessories (glasses, masks, headphones, etc.) in the controlled environment, and (**c**) participants in the wild environment with the annotated face bounding boxes and five-point landmarks.

This paper focuses on face detection and facial landmark detection directly from event streams in controlled and uncontrolled environments.The main contributions of this work are as follows:

- We release an open-source, large-scale, event-based dataset (689 min of recorded event streams) captured under different lighting conditions, from different viewpoints and distances, with multiple people in the scene, and a greater number (73) and diversity of participants (see Figure 1).
- The dataset is fully and accurately labeled with bounding boxes of faces and five-point facial landmarks (eye centers, nose tip, and mouth corners) of all subjects in a scene, releasing over 1.6 million annotated faces in different environments labeled at a rate of 30 Hz.
- We present a dedicated deep learning model for face detection and facial landmark detection. The model, adapted from [20], uses a hybrid architecture combining convolutional and recurrent layers. Our model is the first deep learning model to use recurrent layers for face detection from event streams. It outperforms its well-known frame-based counterpart [21] in accuracy.
- With the open-source data and models, our work can serve as a benchmark for face and facial landmark detection for event-based cameras. We conducted extensive experiments showing real-time face detection from the direct output of event streams.

The rest of the paper is organized as follows: In Section 2, we provide an overview of existing event-based face datasets and state-of-the-art models for face and facial landmark detection from event streams. Section 3 presents our dataset, including the data collection and annotation steps. In Section 4, we explain the underlying model used in the face detection task, as well as enhancements made on the base model to improve the performance and to predict facial landmarks. Information on model training and experimental results are provided in Section 5, and a related discussion can be found in Section 6. We conclude our work in Section 7.

## 2. Related Work

**Figure 2:**
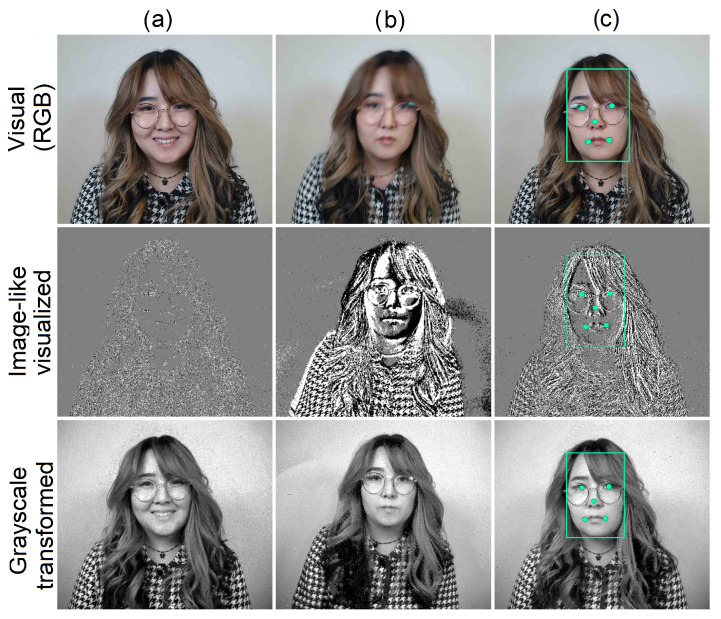
Facial RGB images and the corresponding image-like visualized and grayscale transformed event-based images: (**a**) static pose, (**b**) moving camera causing blur in the visual image, (**c**) facial image with annotated bounding box and five-point facial landmarks. The event-based facial images are generated from the raw event streams using the Metavision software (https://www.prophesee.ai/, accessed on 9 January 2023).

In most applications, face detection and facial landmark detection (see Figure 2c) serve as the first mandatory steps. A substantial amount of research has been conducted in visual [22,23,24], thermal [25,26], depth [27,28], and other domains [29] for face and facial landmark detection. State-of-the-art face detection algorithms rely on deep learning networks [30], particularly convolutional neural networks (CNNs) [31,32], reinforcement learning [33], generative adversarial networks [34], and hybrid architectures [35].

Minaee et al. [36] presented a comprehensive review of face detection methods for conventional cameras, starting with a discussion of early methods, such as Haar cascades classifiers [37] and histogram of oriented gradients (HOG) [38], and moving to more sophisticated approaches presented during the deep learning wave. In addition to a comparison between model architectures, their performance on well-known benchmarks [39,40,41,42,43] was also shown. Deep-learning-based face detection models were classified into the following categories: Cascade CNN-based models, R-CNN-based models, single-shot detector models, feature pyramid network-based models, Transformers-based models, and other architectures. One of the most impressive results was obtained by a single-shot detector called Retina Face [44], which was reported to achieve an average precision (AP) score of 91.4% on the WIDER FACE hard test set.

Data-hungry deep learning models require large datasets for training. Therefore, extensive research has been dedicated to the collection and annotation of face and facial landmark datasets in different domains. WIDER FACE [39] and MegaFace Challenge [45] for the visual domain contain 393,703 and 1 million faces, respectively. Several remarkable RGB face datasets are included in Table 1, along with metrics highlighting the specific features of each dataset. With regard to the thermal domain, the recently presented TFW dataset [46] contains 9982 images of 147 subjects. For depth images, Microsoft Kinect was utilized to create the KaspAROV database featuring 108 subjects recorded on 432 videos [47]. Another depth dataset with extreme head poses called Pandora was presented in [48]. Specifically created for driver pose estimation, it contains 110 annotated sequences with 22 subjects. There are even datasets for artificial faces. For instance, Zheng et al. introduced iCartoonFace, containing 60,000 images with 109,810 cartoon faces [49].

Despite significant advances in face detection in the visual, thermal, and depth domains, to the best of our knowledge, there are only a few face datasets recorded with event-based cameras (see Table 1). The Face Pose Alignment dataset [50] consists of 108 clips with the total duration of 10.2 min acquired during moderate and intense head motion conditions. The eyes and mouth in this dataset are labeled with bounding boxes. The dataset has some drawbacks, such as the low resolution of the sensor (304 × 204), the small number of participants (18), the short duration of the recorded data, and the limited variability of the face poses and camera angles. Another dataset was introduced by Lenz et al. [51] in which the authors recorded event streams of faces for eye blink detection. This dataset contains 50 videos, 25 of which were annotated for face and eye position. However, due to the short duration of the collected videos (13.5 min) and the small number of participants, this dataset does not solve the problem of having a large amount of data to train deep neural networks.

One way to circumvent the dataset problem for event cameras is to synthesize data from traditional frame-based data (RGB or grayscale images) into event-based data, as in [54], but this method may not capture the full advantage of the asynchronous nature of event-based cameras and requires significant computing power for processing. Another popular method for avoiding the problem of adapting computer-vision algorithms is to reconstruct grayscale images from event streams and use deep learning models trained for visual images on the data [54,55,56,57]. The temptation to use this method arises from the availability of standard and well-established computer vision algorithms that become achievable through the grayscale transformation. While the event data offer all the information needed for full-value frame-based image reconstruction [54] (see Figure 2), there is a high computational cost involved in mid-transformations, as well as increased latency issues that are inherent to conventional frame-based images [58].

These issues motivated researchers to use event streams directly for face detection and facial landmark detection. In [51], researchers used event streams directly for real-time face detection using a person’s natural movement (i.e., eye blinking). Since the event camera stops generating events when there is no motion, this method relies on the repetitive nature of eye blinks. Therefore, when the bounding box of the blink of both eyes is detected, the boundaries of the face are determined with an accumulation time of 250 ms. However, this method is sensitive to the camera resolution. Specifically, the model can detect a face at a distance of up to 150 cm from the camera. In a similar study [59], the authors developed the GR-YOLO neural network to obtain the area of the eye blink to determine the area of the face based on facial landmarks.

One of the pioneering methods for direct detection of faces from event streams is described in [60]. Barua et al. used the HOG [38] as input features and utilized a random forest method consisting of 50 trees as a learning algorithm. The results showed that face detection with this method was comparable to the performance of the Viola–Jones detector on original and reconstructed intensity images at a rate of 2000 frames per second. Ramesh et al. [61] also presented a method for face detection using event streams directly. In this work, kernelized correlation filters (KCFs) were used along with an Adaboost classification framework for event-based face detection. The KCFs were reformulated to discriminate between facial and non-facial image areas instead of building upon handcrafted feature descriptors.

There are a number of advantages to performing face-related tasks specifically on event cameras. Apart from the inherent properties of event cameras that make them suitable for working under complex lighting conditions and for dynamic scenes, it was shown in [52] that event cameras are also much better suited for capturing microexpressions of people. Becattini et al. [52] produced an event-reaction dataset and corresponding reaction classifier. Preliminarily, the authors relied on a face alignment tool [62] for RGB images and an open event camera simulator (ESIM) [63] to extract cropped facial data by training a YOLOv3 [64] object detector. Another recent work on facial expression recognition was presented in [53]. In [53], the authors present the NEFER dataset, which consists of paired RGB and event videos containing human faces annotated with face bounding boxes and facial landmarks as well as labeled with the corresponding emotions. The face detector was obtained using the same approach as in [52], except that it was trained on a YOLOv2 [65] object detection model. The authors reported twice the precision accuracy in recognizing seven emotions using an event-based approach compared to detection on RGB videos. In [52], authors highlighted the absence of open-source direct face extractors from event data, and therefore both works [52,53] used alternative methods combining synthetic event data with face alignment to extract facial data before performing their primary tasks.

Overall, our dataset can be applied to a number of event-vision based research tasks, apart from face and facial landmarks detection. The presented dataset can be used standalone or integrated with other event datasets [20] for intensity reconstruction. Another task for which the dataset can serve helpfully is face recognition.

## 3. Faces in Event Streams (FES) Dataset

### 3.1. Experimental Subjects

A total of 73 subjects (31 female and 42 male) participated in the data collection experiments. The average age of the participants was 25.3 years, with a standard deviation of 5.83 years. While 64 subjects participated in data collection in a controlled laboratory environment only, data from nine subjects were collected solely in an uncontrolled (wild) environment. The number of subjects who participated in both experiments was 14. Special attention was paid to the diversity of the participants—that is, inclusion of different ethnicities and individuals with facial accessories (see Figure 1). The study was approved by the Institutional Research Ethics Committee of Nazarbayev University. Each participant signed an informed consent form that allowed the researchers to use the participants’ data to create machine learning models for face and facial landmark detection and to make the dataset public for further research.

### 3.2. Data Collection Experiments

We recorded subjects in versatile conditions to increase the accuracy and generalizability of face and facial landmark detection. Event streams were collected using a Prophesee PPS3MVCD event-based vision system with a spatial resolution of 480×360, a pitch of 30 μm, a temporal resolution of 1 μs, and a field of view of 70°. The event streams were acquired by a desktop workstation (Intel Core i5-8500, 24 GB DDR4 memory, Ubuntu 18.04 Linux operating system) via Universal Serial Bus (USB 3.0) using the Metavision software (https://www.prophesee.ai/, accessed on 9 January 2023).

The four-column event stream data were stored on the hard drive as files with the “.raw” extension.

The experiments can be divided into two major parts based on the controlled and uncontrolled manners of data collection. Detailed information on the specific characteristics of the FES dataset is given in Table 2. Event streams recorded in a controlled manner featuring one participant in a scene are presented under the Lab title, while event-based recordings with multiple people in a view, moving uncontrollably, are referred to as Wild.

Information about the poses of the faces is presented in Table 3. Here, one can see the variability of facial poses in our dataset. Most of the dataset includes facial positions where the face is fully visible, i.e., the frontal face. Nonetheless, considerable attention was paid to diverse face detection angles, such as the face profile position and face angles captured at an approximately 3/4 angle view, as well as vertical movements and their respective positions.

**Figure 3:**
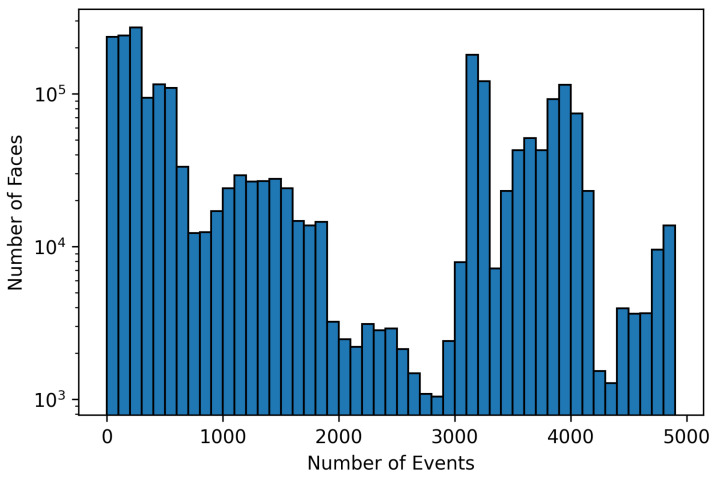
Histogram of faces by event numbers during the 50 ms accumulation period.

Another factor describing our event-based dataset is the variability in the presence of motions. Figure 3 displays a histogram of event counts obtained from the facial area, with the aim of filtering out noise from the surrounding regions. This extraction process was carried out using event streams with a 50 ms accumulation period. As can be seen from the figure, the dataset contains both slow movements of the face, which are barely detected by the event sensor, and fast movements, which are detected from 3000 event points and more (see Figure 3).

Data collection in the controlled experiments was conducted in an indoor environment under bright and dim lighting conditions, at 50, 150, and 400 cm from the camera, with the camera either moving or kept fixed. Head postures and movements included left–right, up–down, and circular movements of the head and counting, while walking patterns consisted of zigzag walking, walking toward the camera, and sideways walking. For each subject, 60 recordings were obtained.

Data in the uncontrolled condition were also collected indoors (e.g., in hallways, a coffee shop, and a classroom on the university campus). In these experiments, groups of 10 to 12 subjects were asked to move freely within the field of view of the event camera. As there were no specific instructions on the movements and head postures, subjects behaved casually during the recording process and wore various accessories (e.g., hats, masks, or glasses).

### 3.3. Data Annotation and Curation

**Figure 4:**
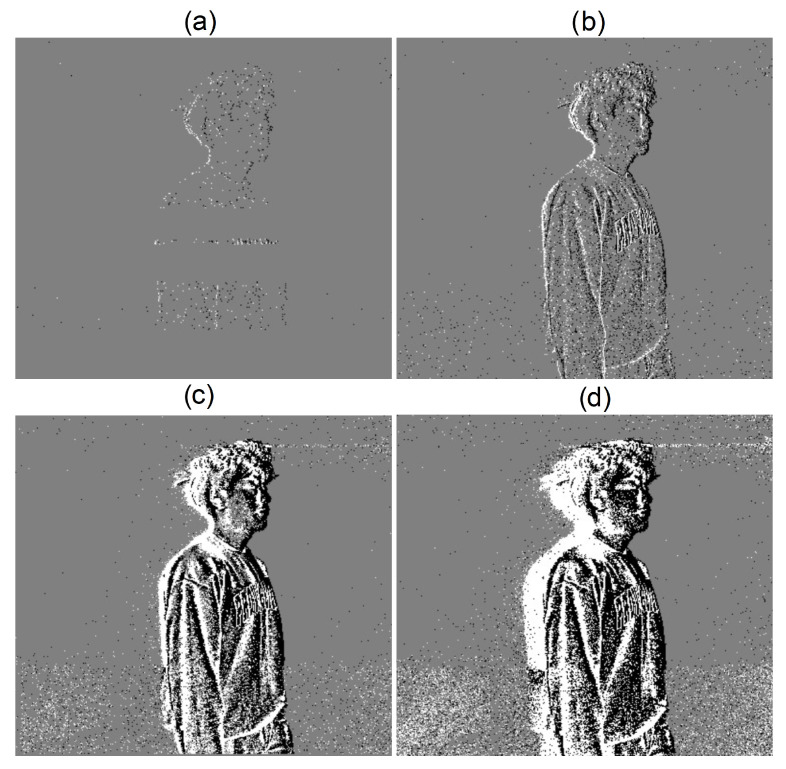
Image-like visualization of event streams with different accumulation times: (**a**) 0.2 ms, (**b**) 1 ms, (**c**) 20 ms, and (**d**) 100 ms. The event streams were rendered by defining ON events as white pixels, OFF events as black pixels, and the background as gray.

It is difficult for a human to infer the spatial representation of a scene from an event stream. Therefore, an image-like visualization of event streams is obtained by accumulating events over a short period of time (the accumulation time) and visualizing the ON and OFF events on an image with different colors (usually black and white) using the spatial coordinates of the events (see Figure 4).

In this work, we used this technique as a preprocessing step for annotation with a 33 ms accumulation time. The number of images in Table 2 refers to these visualizations. In the resulting 1.24 million images, we annotated the face bounding boxes and the five-point facial landmarks (see Figure 1c). The annotation task was performed using the open-source toolkit CVAT (https://cvat.ai, accessed on 9 January 2023) by four annotators over a period of nine months. The quality of the annotations was regularly checked by the first author of the paper.

**Figure 5:**
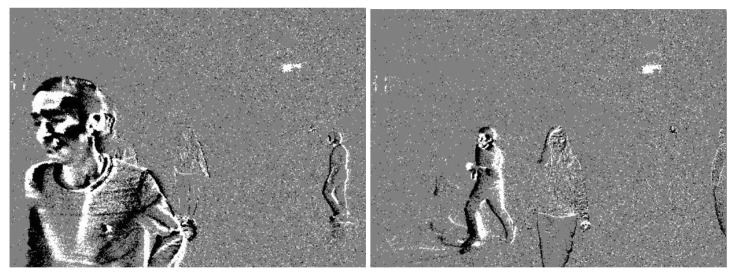
Blurry facial observations in the wild setup.

To properly handle cases where there was no motion or activity, resulting in the absence of facial observations, and to clearly identify facial boundaries during fast movement, a special protocol was provided for the team that performed the annotation process. To increase the precision of the annotations in extreme settings, two types of videos were used for face and facial landmarks labeling. In good lighting conditions and slow/average motion, grayscale videos with a frame rate of 30 frames per second were used for annotation. In low light conditions and fast movements, event-based original recordings were used and annotated at the same frame rate. This made it possible to minimize the cases where no facial image could be extracted from the frames. Given the continuous movement of the subjects, where sufficiently clear face images were occasionally not available, it was still visually possible to extract the face and facial landmark features between two evident frames of the subjects. Nevertheless, a smaller portion of frames (less than 0.2%) was skipped when no facial feature information could be extracted to keep the dataset clean. As for fast movements, 33 ms is a relatively short period of time for a human to move substantially. The blurry facial boundaries that result from fast movements were not significant enough to degrade the facial observation. Samples of blurry facial observations in the wild environment during fast movements can be found in Figure 5. It can be seen that the facial boundaries are clearly visible despite the movement speed.

For machine learning purposes, both raw and preprocessed datasets were divided into training (Train), validation (Valid), and testing (Test) sets, as shown in Table 2. Specifically, the training, validation, and testing sets contain 60%, 22%, and 18% of the dataset, respectively. In the controlled part of the dataset, the data were split based on subject identifiers (IDs), i.e., a subject appears in one of the sets only.

To evaluate the accuracy of determining the boxes bounding faces, faces were divided into three groups depending on the distance of the face from the camera—large, medium, and small, as shown in Table 4. Large faces were considered as those whose bounding box height was greater or equal to 90 pixels; medium faces were those whose height was between 35 and 90 pixels; and small faces were of a height below 35 pixels.

## 4. Deep Faces in Event Streams (DFES) Model

In this section, we describe the architecture of our Deep Faces in Event Streams (DFES) model for face bounding box and five-point facial landmark detection. Our model is based on the model in [20] used for object detection in event streams. In this model, the researchers introduced a mechanism in which learning occurs through incoming events accumulated over a period of time and through the use of information from past events.

In our model, an event stream is represented as a sequence of events $E=\{e_{j}=\left(x_{j},y_{j},p_{j},t_{j}\right)\}$, where $x_{j}\in \left[0,K-1\right]$ and $y_{j}\in \left[0,L-1\right]$ are the pixel coordinates of an event with the polarity $p_{j}\in \left[0,1\right]$. $t_{j}\in \left[0,\infty \right]$ is the timestamp of the event $e_{j}$. The faces are denoted using axis-aligned bounding boxes $F_{bb}=\left\{f_{bb}=\left(t,x,y,w,h\right)\right\}$, where *x* and *y* are the coordinates of the lower left pixel and *w* and *h* are the width and height of a bounding box, and *t* is the time at which a face is present in a scene. Similarly, five-point facial landmarks are denoted as a sequence of ordered points corresponding to the eyes, nose, and the left and right corners of the mouth, $F_{fl}=\left\{f_{fl}=\left(t,x_{e1},y_{e1},x_{e2},y_{e2},x_{n},y_{n},x_{m1},y_{m1},x_{m2},y_{m2}\right)\right\}$.

The face detection problem can be formally defined as a mapping from *E* to $F_{bb}$ and written as $F_{bb}=D_{bb}\left(E_{ti<t}\right)$, where the detector $D_{bb}$ should predict face bounding boxes using past events. Likewise, the combined detection of faces and facial landmarks can be defined as $\left(F_{bb},F_{fl}\right)=D_{bb+fl}\left(E_{ti<t}\right)$.

**Figure 6:**
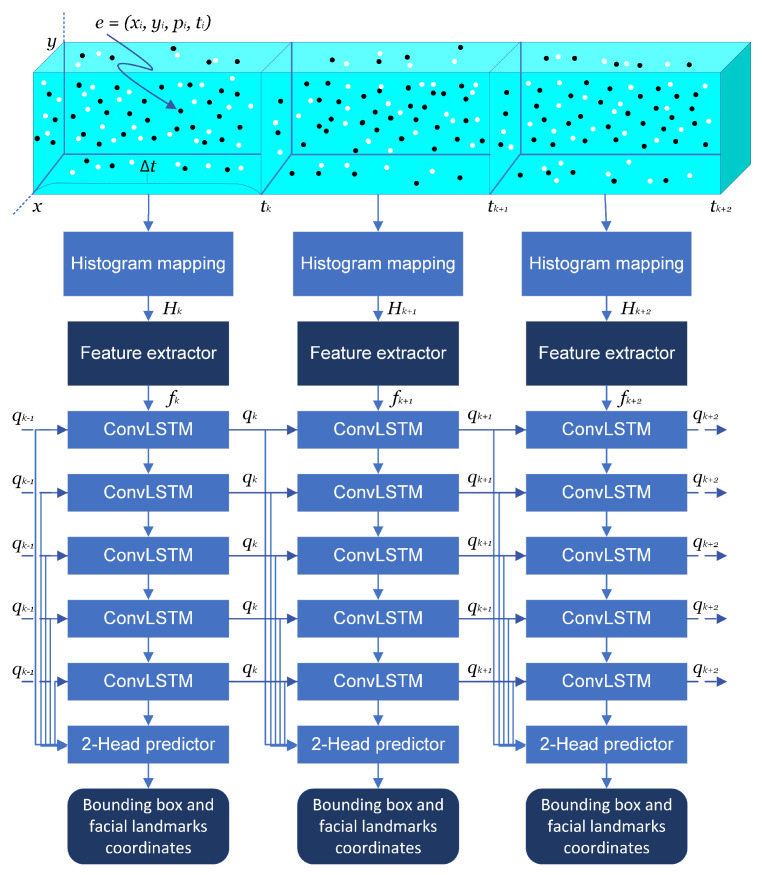
DFES model architecture for face detection and facial landmark extraction (adapted from [20]), where $q_{0}=0$.

In this work, the detectors $D_{bb}$ and $D_{bb+fl}$ are implemented using deep learning models (see Figure 6). Since applying the detector *D* to all past events is computationally intractable, incoming events during the period $\left[t_{k},t_{k+1}\right]$ are collected in an array for the period $\Delta t=t_{k+1}-t_{k}$. This array is then transformed into a tensor map $H_{k}$ using the histogram preprocessing function Hist(.) to group each event during Δt in the corresponding cells, depending on the *x* and *y* coordinates of the pixels and their polarity. The resulting tensor map $H_{k}$ has the dimensions 2×K×L, where *K* is the width and *L* is the height of the tensor map.

Afterward, the features $f_{k}$ are extracted from the tensor map $H_{k}$. In [20], squeeze-excite layers [66] were utilized to obtain spatial information from the features. In our work, we also used the residual neural network (ResNet) architecture [67] for feature extraction. The advantages of the ResNet feature extractor [67] (primary components, residual blocks, and connection skipping) served as the motivation for our work to test it. The key advantage of this network is the use of the residual block, which binds two convolutional layers using a skip connection. Specifically, we incorporated the ResNet-18, ResNet-34, and ResNet-50 variants in our network. The architecture and parameters of the feature extractors used for the DFES model are summarized in Table 5.

To exploit prior events, the detectors $D_{bb}$ and $D_{bb+fl}$ depend not only on the events accumulated in the present Δt, but also on the encoded information from the past stored as an internal state $q_{k-1}$ in $D_{\left(.\right)}={D_{\left(.\right)}\left({\left[e_{j}\right]}_{\Delta t},q_{k-1}\right)}_{k}$. A recurrent neural network architecture was utilized to generate the internal state vector $q_{k}$. Therefore, the output of the feature extractor was connected to a five-layer convolutional long short-term memory (ConvLSTM) network [68] that obtains the internal state information $q_{k-1}$ in addition to the extracted features $f_{k}$. Finally, the output of each ConvLSTM layer is fed into a two-head regression predictor consisting of convolutional layers to predict face detection bounding boxes and five-point facial landmarks, as implemented in [20]. Thus, this two-regression-head predictor detects objects at multiple scales. To achieve this, our model first supplies feature maps of differing scales to the feature extractor and ConvLSTM layers. Each scaled feature map from the ConvLSTM layers is then fed into both regression branches and used as input to the predictor.

To train our neural network, we used the cost function $L_{t}=L_{reg}+L_{cls}$, where $L_{reg}$ was a smooth l1 loss [69] for regression and $L_{cls}$ was a softmax focal loss [21] for classification (between the background and face classes). Our implementation of the softmax focal loss is identical to that in [20]. To describe the smooth l1 loss in our model, let $F_{bb}^{*}$ and $F_{fl}^{*}$ denote the ground truth values for the face bounding boxes and five-point facial landmarks, respectively. Similarly, $F_{bb}^{\prime }$ and $F_{fl}^{\prime }$ denote the predicted versions. If we combine the bounding box and facial landmark values for the ground truth and predicted values as $F_{gt}=\left\{F_{bb}^{*},F_{fl}^{*}\right\}$ and $F_{pr}=\left\{F_{bb}^{\prime },F_{fl}^{\prime }\right\}$, we can write the smooth l1 loss function as follows:

(1) $$\begin{array}{cc} L_{reg}\left(F_{gt},F_{pr}\right) & =\frac{1}{N}\sum_{i}L_{reg}\left(F_{gt}^{i},F_{pr}^{i}\right)\\ L_{reg}\left(F_{gt}^{i},F_{pr}^{i}\right) & =\left\{\begin{array}{cc} 0.5{\left(F_{pr}-F_{gt}\right)}^{2}/\beta & \mathrm{if}\;(F_{pr}-F_{gt})<\beta \\ F_{pr}-F_{gt}-0.5\beta & \mathrm{otherwise}\; \end{array}\right. \end{array}$$

where β is a tunable parameter to control the contributions of the losses L1 (mean absolute error loss) and L2 (mean squared error loss). In this work, this value is the same as in [20], i.e., β = 0.11.

## 5. Experiments

### 5.1. Model Training and Testing

The designated training set was used to train neural network models, while the validation event streams were used to tune the hyperparameters of the network. Next, the accuracy of predicting face bounding boxes and landmarks was evaluated on testing set event streams.

For model training, we used a Nvidia DGX-2 server with V100 graphics processing units (GPUs). All the models were trained from scratch. We implemented the models in PyTorch and enabled the multiprocessor parallel model training mode in the PyTorch tool. Each model was trained using the Adam optimizer with a learning rate of 0.0001 for 40 epochs. Training took about two days for each model. After each epoch, the resulting model was saved as a checkpoint. Once the training was completed, the best checkpoint model (based on the validation set performance) was employed for testing. A total of 12 models were trained. Six models were trained using the original architecture [20] with the combinations of three different accumulation times (33 ms, 50 ms, and 100 ms) and two models for bounding box detection (DFES_BB_) and both facial landmark and bounding box detection (DFES_FL+BB_). The main purpose here was to analyze the effects of accumulation time on model performance. Based on the results of this analysis, the best accumulation time was chosen for further experiments. Consequently, six additional models were trained by replacing the original feature extractor from [20] with ResNet variants (ResNet-18, ResNet-34, and ResNet-50) for the two cases (BB and FL + BB). Table 5 lists the feature extractors used in the study. These models were tested on unseen event streams to determine the coordinates of the face bounding box and facial landmarks. The main objective of this experiment was to explore the effects of different feature extractors on model performance.

Event cameras are usually employed on edge computing nodes and in dynamic environments. The model inference time is of paramount importance for deployability in real-world applications. Therefore, we also measured the inference time of the models. This also affects the latency of the face bounding box and landmark detection. To support the motivation for the usage of direct event streams models for face and facial landmark detection tasks, we also conducted an experiment that compared the performance of DFES with a popular frame-based detector (RetinaNet). Finally, the DFES model was tested in real time using an EVK-4-HD event-based camera connected to a workstation (Intel Xeon L3403, 64 GB DDR3 memory, and Nvidia GeForce RTX 2080 Ti GPU) with the Windows 10 operating system to prove the real-time operation of our machine learning pipeline.

To evaluate the accuracy of the predicted face bounding boxes, we employed the mean average precision (mAP) measure at an intersection over the union (IoU) threshold of 0.5, also known as the mAP_50_ metric. Additionally, to measure the accuracy of facial landmark prediction, we employed the normalized mean error (NME) metric, similar to the approach in [62]. This metric factors in the size of the ground box and considers facial movements across the horizontal and vertical planes:

(2) $$NME=\frac{1}{N}\sum_{p=1}^{N}\frac{{\hat{x}}_{p}-x_{p}}{D_{p}}$$

where *N* is the number of faces, ${\hat{x}}_{p}-x_{p}$ is the Euclidean distance between the predicted ${\hat{x}}_{p}$ and the ground truth $x_{p}$ landmarks, and $D_{p}$ is the square root of the width and height of the ground truth bounding box, denoted as $D_{p}=\sqrt{wh}$.

### 5.2. Models with Fixed Number of Events

In addition to the main windowing strategy based on accumulation time, another methodology of slicing based on a fixed number of events was applied to evaluate the efficacy of the different windowing methods. Using trial and error, 137,000 events were selected to build an image-like visualized frame and utilize it as a slicing mechanism for model training. The identical setup, model architectures, and evaluation metrics utilized for accumulation time-based algorithms encompassing bounding box and facial landmark detection were employed to build models maintaining a constant number of events as well. Specifically, the DFES_BB+FL_ model was trained with four feature extractors consisting of the original and variations of ResNet (ResNet-18, ResNet-34, and ResNet-50).

## 6. Results and Discussion

### 6.1. Determination of the Optimal Accumulation Time

To determine the best accumulation time, we trained the model with different accumulation times. Table 6 shows the bounding box detection results for the DFES_BB_ and DFES_BB+FL_ models with the original feature extractor for 33 ms, 50 ms, and 100 ms on the testing set.

The best mAP_50_ results were obtained using a model trained on event streams with an accumulation time of 50 ms and an overall mAP_50_ equal to 0.95 for the bounding box detection model and 0.918 for the combined facial landmark and bounding box detection model. The accumulation time of 33 ms is likely to have been too short a period to collect a sufficient number of events for face detection. Another possible explanation is that the accumulation time of 33 ms is approximately 1.5 times shorter than the 50 ms accumulation time. As a result, the model with an accumulation time of 33 ms processes more frames within a given duration than the model with an accumulation time of 50 ms. This higher number of frames may result in a larger number of testing samples during the evaluation, which in turn may impact the mAP score. The larger number of testing samples can be challenging to evaluate the performance of the model. It introduces greater variability in predictions and can potentially result in lower mAP scores. With a larger number of frames, matching the model predictions to the ground truth annotations becomes more challenging, making it harder to achieve high precision and recall values. Similarly, the longer accumulation time of 100 ms might have collected an excessive number of events on the face region, causing an equivalent of the blur effect in the visual images for the event stream. Therefore, an accumulation time of 50 ms was used for further experiments with event streams. As was stated earlier, to investigate the effects of various accumulation duration on the functionality of the event-based camera, we used accumulation lengths of 33 ms, 50 ms, and 100 ms in our study. While this approach imposes limitations as test sets vary by accumulation times, it provides valuable insights into the behavior of the system under different event accumulation times.

### 6.2. FL and BB Detection Results

In this experiment, the DFES models were trained using various ResNet-based feature extractors (see Table 5). For granular benchmarking of the model performance, the test data were divided into laboratory, wild, and overall sets. In addition, detection results were obtained for large, medium, small, and overall (all) faces.

Table 6, Table 7 and Table 8 present the mAP_50_ results for bounding box detection and the NME results for facial landmark detection, respectively. In general, the mAP_50_ score was higher for large faces, showing the difficulty of detecting faces that were further away from the camera. Also, compared to the laboratory testing set results, the results for the wild part of the data received a lower score. This might have been caused by the presence of multiple faces on event streams in uncontrolled poses.

The best results for face bounding box detection were achieved by the model with the ResNet-18 feature extractor and an accumulation time of 50 ms (see Table 6). This model achieved a score of 0.978 mAP_50_ on the laboratory testing set, 0.440 on the wild testing set, and 0.895 mAP_50_ on the whole testing set.

Comparing DFES_BB_ model performance and previous direct detection of facial bounding boxes from event streams, a model from [60] combining HOG with random forest achieved 67% accuracy on simulated event data, and KCFs with Adaboost [61] within 90–10% train–test split reached 84.42% accuracy on DAVIS dataset recorded on a camera with 240 × 180 resolution; the DFES_BB_ model presented in this work showed 97.8% accuracy on the FES dataset, thus expanding the performance limits of direct face detectors from event streams.

**Figure 7:**
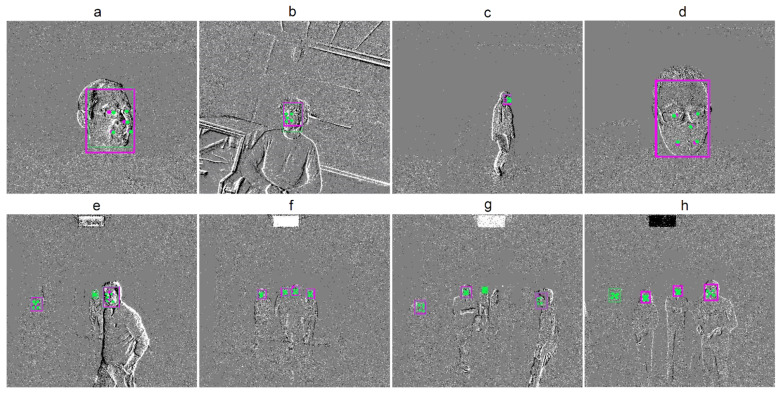
Samples of the predicted versus the ground truth bounding box and facial landmarks for the model with the ResNet-34 feature extractor from the controlled (**a**–**d**) and wild (**e**–**h**) environments. The green color denotes the ground truth, and the magenta color denotes the predictions.

Among the combined facial landmark and bounding box detection DFES_FL+BB_ models, we obtained the highest mAP_50_ score on the wild testing set using a model with an accumulation time of 100 ms and the original feature extractor (see Table 7). Nevertheless, the best overall results were achieved using the model with the original feature extractor and an accumulation time of 50 ms. Specifically, this model had the following mAP_50_ scores: 0.973 on the laboratory part of the testing set, 0.528 on the wild part, and 0.918 on the overall testing set. Figure 7 illustrates some testing samples processed by this model. The model detects the bounding box and the landmarks of the face, but there are discrepancies between the predicted results and the ground truth. Even on the wild testing set, some faces, especially small ones, were not detected, although the model predicted most faces accurately. It is worthwhile to note that the DFES_FL+BB_ model showed higher performance than the DFES_BB_ model in the wild and, in some cases, in the laboratory for bounding box detection and, consequently, in the overall testing set. Presumably, facial landmarks contributed to the performance of bounding box detection as an additional source of information that guided the model training process.

**Figure 8:**
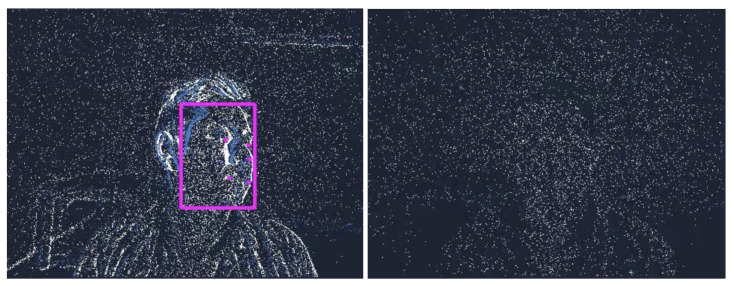
DFES_FL+BB_ model performance on a moving subject (**left**) versus a stationary subject (**right**). The face of the moving subject is detected correctly, whereas the face is not detected when the subject is stationary.

Figure 8 demonstrates the contrast between face detection results of the DFES_FL+BB_ model on moving and stationary subjects. Event cameras are particularly efficient and can exhibit superior performance in comparison with conventional cameras for fast-moving objects and poor lighting conditions. However, one should keep in mind that for stationary objects, detection results might be of low quality due to the nature of event cameras that rely on events due to pixel intensity changes.

Table 8 summarizes the facial landmark prediction results. The lowest NME score (4.3) on the whole testing set was obtained using the model with an accumulation time of 50 ms and the ResNet-50 feature extractor. In contrast, the model with 33 ms accumulation time achieved the worst NME score (6.41). It might be the case that a lower number of events is insufficient for accurately predicting facial characteristics. Despite the fact that the model with a ResNet-50 feature extractor had more errors in predicting facial landmarks in the lab test set, it demonstrated the lowest scores on the wild test for all faces. In comparison, the lowest NME score on overall laboratory testing set was obtained using the ResNet-34 feature extractor model. Also, the overall NME results on the test set were somewhat closer to the laboratory results. This is primarily due to the proportion difference in size between laboratory and wild testing sets. The laboratory test set is 85.08% out of the overall set, which is significantly larger than the wild set, which can influence the overall NME scores and lead to the observed variations in comparison to the wild test set (see Table 2). In Figure 7, we can see that the model predicted facial landmarks better in the laboratory portion of the testing set, but, similar to the bounding box case, there was a small difference between the predicted and ground truth facial landmarks.

### 6.3. Comparison with Frame-Based Model

RetinaNet [21] is a popular frame-based camera deep learning model. It uses a novel architecture that combines the strengths of two critical components: a backbone network and a feature pyramid. This network was customized to process the frames generated from event streams and named Events-RetinaNet [20]. Compared to DFES models, this model does not have a temporary memory, which can lead to loss of detection after a short stop in motion.

In this study, we trained the Events-Retina model from scratch for bounding box detection using our training data for 40 epochs. This model achieved a score of 0.865 mAP_50_ on the overall test set. As can be seen in Table 6, our model with ResNet-18 as a feature extractor achieved better results than the Events-RetinaNet model, with a higher mAP_50_ score of 0.895. This result highlights that models with direct event stream inputs are advantageous compared to frame-based methods.

### 6.4. Windowing Strategy with Fixed Number of Events

To explore the nature of the accumulation of event streams and their behavior based on different slicing techniques, we also experimented with a windowing strategy using a fixed number of events. The same deep learning architecture with the same initial set of annotated faces yielded worse results when the accumulation was based on the number of events instead of the temporal dimension, reporting an mAP_50_ score with original extractor 0.45, with ResNet-18 feature extractor 0.46, with ResNet-34 extractor 0.46, and with ResNet-50 feature extractor 0.28. Overall, it was concluded that for the fixed number of events, widowing strategy annotations at the corresponding rate should be developed.

### 6.5. Inference Time and Real-Time Detection Experiment

In this experiment, we measured the inference time of face bounding box and facial landmark detection by the model for a single frame on a single GPU (Tesla V100). We found that as the accumulation time increased, the time taken to predict bounding box and facial landmark coordinates in one frame increased slightly. Specifically, the models with accumulation times of 33 ms, 50 ms, and 100 ms predicted the testing data outputs in 10.3 ms, 10.5 ms, and 10.7 ms, respectively. It should also be noted that models whose input histogram maps were created with an accumulation time of 33 ms processed more frames than those with accumulation times of 50 ms and 100 ms. Processing more frames increases the computation load.

The model with the ResNet feature extractor had a longer inference time than the original model. Thus, the ResNet-18 feature extractor model predicted faces in 11.9 ms, the ResNet-34 feature extractor model in 13.5 ms, and the ResNet-50 feature extractor model had an inference time of 15.83 ms (see Table 9). We observed no significant change in inference time between the DFES_BB_ and DFES_FL+BB_ models.

For each case, the inference time was shorter than the event accumulation time, demonstrating the possibility of using models for real-time face detection. In the context of real-time deployability, the aspects of model architecture, such as size of the model and its associated memory requirements, plays a significant role in determining the needed hardware. Thus, the different model configurations, along with their corresponding numbers of learnable parameters and memory sizes, are provided in Table 9. It should be mentioned that the Nvidia GeForce RTX 2080 Ti GPU on the desktop workstation could run models in real time without any problems. Moreover, these models with the specified memory requirements in Table 9 can even run on edge AI devices such as (NVIDIA Jetson) with 4 GB of memory.

## 7. Conclusions

Event cameras, a new type of retinomorphic sensors, have advantages over conventional frame-based cameras and find various applications in computer vision. However, due to the relative novelty of such cameras, there are only few datasets to apply deep learning to event streams, which limits the application of these bioinspired sensors.

To address this problem, we prepared the FES dataset. To the best of our knowledge, this is the first large and diverse event-based camera dataset for face detection. The FES dataset contains 689 min of raw event streams recorded in wild and controlled environments with multiple face poses and distances. In addition, the dataset contains accurately annotated bounding box and facial landmark coordinates. Thus, the dataset is already prepared for the application of machine learning and other algorithms.

To validate the efficacy of our dataset, we trained six models for bounding box detection only and another six models for simultaneous detection of face landmarks and the coordinates of bounding boxes. The models were able to detect the faces and facial landmarks accurately, showing the usefulness of the FES dataset. The models can also be run in real time. To stimulate further research in this area, we share our dataset, codes, and trained models at https://github.com/IS2AI/faces-in-event-streams, accessed on 2 December 2023 under MIT license.

Direct event-based face extraction might serve as a solid base for further face-related tasks such as emotion classification or face recognition. As previously conducted works [52,53] have demonstrated the superiority of the neuromorphic event-based approach over RGB-based models for emotion and microexpression recognition, this implies the richness of features obtained from event-based facial representations. The current study showed better performance of the DFES models in comparison with the Event-RetinaNet model, which relies on frame-based representations of event streams. Nonetheless, to fully prove the efficiency of direct event-based face detection over RGB-based face detection models, a separate comparison study on event streams paired with RGB frames should be conducted to investigate the matter in more broad terms. Additionally, in terms of future work, we will focus on increasing the size of the dataset and improving the performance of the models by employing data augmentation and new deep network architectures. We also intend to expand upon this research topic by leveraging the presented dataset for face recognition tasks involving event streams.

## Figures and Tables

**Table 1:** Face and object datasets acquired with event and RGB cameras.

Dataset	Camera	Purpose	Env.	Constrained	# Images	Length (min)	Subject	Resolution	BBox	LM	# Labels
WIDER FACE [39]	RGB	FD	in, out	wild	32,203	–	–	1024 × 754	✓	–	393,703
Pascal Face [40]	RGB	FD	in, out	wild	851	–	–	1280 × 720	✓	–	1341
MALF [41]	RGB	FD	in, out	wild	5250	–	–	363 × 450	✓	–	11,931
UFDD [42]	RGB	FD	in, out	wild	6425	–	–	301 × 1024	✓	–	5171
VGGFace2 Dataset [43]	RGB	FD	in, out	wild	3.31 M	–	9131	137 × 180	✓	–	3.6 M
1Mpx Automotive Detection Dataset [20]	event	OD	out	wild	–	840	–	1280 × 720	✓	–	25 M
Face Pose Alignment [50]	event	FD	in	controlled	–	10.22	18	304 × 204	–	3	–
Lenz et al. [51]	event	FD	in, out	controlled	–	13.5	10	640 × 480	–	2	–
Event-reaction Dataset [52]	event	ER	in	controlled	–	∼75.8	25	640 × 480	✓	–	–
NEFER [53]	event	MER	in	controlled	–	∼73.5	29	1280 × 720	✓	68	–
FES (ours)	event	FD	in	controlled, wild	–	689	73	480 × 360	✓	5	1.6 M

Note. FD: face detection, OD: object detection, ER: emotion recognition, MER: micro-expression recognition, in: indoors, out: outdoors, BBox: bounding box, LM: landmarks.

**Table 2:** Statistics of the FES dataset.

Category	Train		Valid		Test		Total		
	Lab	Wild	Lab	Wild	Lab	Wild	Lab	Wild	Both
# of subjects	40	15	12	14	12	11	64	18	73
# of images (thousands)	720.0	57.3	216.0	19.1	216.0	11.0	1152.0	87.4	1239.4
# of labeled faces (thousands)	715.4	357.9	210.5	82.4	215.0	37.7	1140.9	478.0	1618.9
# of recordings	2400	32	720	10	720	7	3840	49	3889
Duration (min)	400	28	120	10	120	11	640	49	689
Mean duration per record (s)	10	60	10	63	10	52	10	59	10.62
Standard deviation of duration per record (s)	0.5	6.2	0.4	2.7	0.5	19.0	0.5	9.1	5.6
Mean # of events per record (millions)	28.5	155.5	28.7	195.2	29.7	193.3	28.7	169.0	30.5
Standard deviation # of events per record (millions)	12.7	85.5	12.5	35.0	13.5	76.3	12.8	77.6	21.9
Mean # of ON events per record (millions)	15.7	90.8	16.1	104.9	16.6	110.6	16.0	97.8	17.3
Standard deviation # of ON events per record (millions)	6.9	48.3	6.9	31.5	7.5	43.7	7.0	45.2	13.6
Mean # of OFF events per record (millions)	12.5	69.6	12.6	78.6	13.1	82.7	12.6	74.3	13.6
Standard deviation # of OFF events per record (millions)	5.6	33.8	5.6	23.8	6.0	32.6	5.7	32.1	10.3

Note: The sum of the number of subjects in the lab and wild conditions does not equal the total number of subjects, as some subjects were recorded for both sets of experiments.

**Table 3:** Face orientation statistics for the FES dataset.

Dataset	Wild (%)	Laboratory (%)	Overall (%)
Frontal Face	57.01	63.00	62.60
Profile Face	12.20	10.96	11.00
Appr. 3/4 View	30.48	22.00	22.65
Upward Face	0.16	2.67	2.48
Downward Face	0.30	1.29	1.22

**Table 4:** Face bounding box size statistics for the FES dataset.

	<35 pixels	35–90 pixels	≥90 pixels
Wild	0.2%	7.9%	91.9%
Laboratory	41.1%	29.9%	29.0%
Overall	39.5%	29.1%	31.4%

**Table 5:** Feature extractors for DFES network.

Original	ResNet-18	ResNet-34	ResNet-50
ConvLayer, 32	ConvLayer, 16	ConvLayer, 16	ConvLayer, 16
BatchNorm	MaxPool	MaxPool	MaxPool
ReLU	[Resblock, 16] × 2	[Resblock, 16] × 3	BatchNorm,
Squeeze-excite block	[Resblock, 32] × 2	[Resblock, 32] × 4	ReLU
Squeeze-excite block	[Resblock, 64] × 2	[Resblock, 64] × 6	[Bottleneck, 16] × 3
–	[Resblock, 128] × 2	[Resblock, 128] × 3	[Bottleneck 32] × 4
–	–	–	[Bottleneck 64] × 6
–	–	–	[Bottleneck 128] × 3

**Table 6:** DFES_BB_ results of the face bounding box detection models on the FES testing set that can only detect a bounding box.

Feature Extractor	Δt	mAP_50_ Lab Test Set				mAP_50_ Wild Test Set				mAP_50_ Overall Test Set			
		Large	Medium	Small	Overall	Large	Medium	Small	Overall	Large	Medium	Small	Overall
Original	33 ms	0.375	0.400	0.328	0.353	0.417	0.249	0.136	0.239	0.375	0.382	0.288	0.329
Original	50 ms	**0.990**	**0.978**	**0.970**	**0.978**	0.558	0.534	0.312	0.430	**0.989**	0.939	0.740	0.880
Original	100 ms	0.988	0.973	0.964	0.977	0.474	0.430	0.274	0.304	0.987	0.925	0.738	0.876
ResNet-18	50 ms	**0.990**	0.974	**0.970**	**0.978**	**0.693**	0.593	0.348	**0.440**	**0.989**	**0.948**	**0.763**	**0.895**
ResNet-34	50 ms	0.989	0.962	0.952	0.965	0.687	**0.663**	**0.389**	**0.440**	**0.989**	0.947	0.751	0.892
ResNet-50	50 ms	0.987	0.964	0.900	0.957	0.173	0.159	0.060	0.120	0.987	0.888	0.596	0.810

Note. The highest accuracy within each face category is shown in bold.

**Table 7:** DFES_FL+BB_ results of the face bounding box detection models on the FES testing set that can detect both facial landmarks and bounding boxes.

Feature Extractor	Δt	mAP_50_ Lab Test Set				mAP_50_ Wild Test Set				mAP_50_ Overall Test Set			
		Large	Medium	Small	Overall	Large	Medium	Small	Overall	Large	Medium	Small	Overall
Original	33 ms	0.371	0.397	0.380	0.370	0.599	0.443	0.260	0.252	0.369	0.393	0.325	0.347
Original	50 ms	0.989	**0.978**	**0.871**	**0.973**	0.728	**0.782**	0.482	0.528	0.989	**0.970**	0.700	**0.918**
Original	100 ms	0.989	0.976	0.700	0.937	0.640	0.700	**0.645**	**0.653**	0.989	0.949	0.575	0.868
ResNet-18	50 ms	**0.990**	0.969	0.800	0.960	0.720	0.750	0.470	0.500	**0.990**	0.960	0.700	0.900
ResNet-34	50 ms	**0.990**	**0.978**	0.869	0.966	**0.789**	0.750	0.498	0.540	**0.990**	**0.970**	**0.720**	0.912
ResNet-50	50 ms	0.985	0.928	0.750	0.925	0.184	0.282	0.124	0.138	0.984	0.873	0.520	0.800

Note. The highest accuracy within each face category is shown in bold.

**Table 8:** NME results of the bounding box and facial landmark detection models DFES_FL+BB_ on the FES testing set.

Feature Extractor	Δt	NME Lab Test Set				NME Wild Test Set				NME Overall Test Set
		Large	Medium	Small	Overall	Large	Medium	Small	Overall	
Original	33 ms	1.59	3.90	14.69	6.35	7.97	15.50	20.22	19.36	6.41
Original	50 ms	**0.82**	1.44	5.90	2.44	7.25	15.30	20.50	19.40	4.88
Original	100 ms	1.18	1.95	**2.52**	1.80	8.30	15.19	20.97	19.60	4.57
ResNet-18	50 ms	0.90	**1.39**	9.11	3.47	8.24	15.66	22.89	21.14	6.02
ResNet-34	50 ms	0.88	1.91	2.76	**1.71**	8.26	15.68	21.18	20.10	4.36
ResNet-50	50 ms	0.94	3.00	4.37	2.34	**7.10**	**13.30**	**20.00**	**16.50**	**4.30**

Note. The highest accuracy within each face category is shown in bold.

**Table 9:** Statistics of the complexity of the experimented DFES models.

	Number of Learnable Parameters	Memory Size	Inference Time
DFES_BB_ (Original)	24.1 M	3224 Mb	10.50 ms
DFES_BB_ (ResNet-18)	24.4 M	3236 Mb	11.90 ms
DFES_BB_ (ResNet-34)	25.8 M	3249 Mb	13.50 ms
DFES_BB_ (ResNet-50)	64.9 M	3287 Mb	15.83 ms

## Data Availability

The data presented in this study are openly available at https://doi.org/10.48333/fagy-tb79, accessed on 2 December 2023.

## Abbreviations

The following abbreviations are used in this manuscript:

FES	Faces in Event Streams
DFES	Deep Faces in Event Streams
IoT	Internet of Things
FFA	Fusiform face area
CNN	Convolutional neural network
HOG	Histogram of oriented gradients
KCF	Kernelized correlation filters
ESIM	Event camera simulator
NME	Normalized mean error
IoU	Intersection over the union
mAP	Mean average precision
FL	Facial landmark
BB	Bounding box
